# Adapting and validating the Autism Diagnostic Observation Schedule Version 2 for use with deaf children and young people

**DOI:** 10.1007/s10803-021-04931-y

**Published:** 2021-03-24

**Authors:** Helen Phillips, Barry Wright, Victoria Allgar, Helen McConachie, Jennifer Sweetman, Rebecca Hargate, Rachel Hodkinson, Martin Bland, Hannah George, Anna Hughes, Emily Hayward, Victoria Fernandez Garcia De Las Heras, Ann Le Couteur

**Affiliations:** 1 Leeds and York Partnership NHS Foundation Trust, Leeds, UK; 2 Hull York Medical School, University of York, York, UK; 3 Newcastle University, Newcastle, UK; 4 South West London and St George’s NHS Trust, London, UK

**Keywords:** Deaf, Autism Spectrum Disorder, Assessment, Delphi consensus, Child, Diagnosis, Sign language, Autism diagnostic observation schedule

## Abstract

We report a Delphi Consensus modification and first validation study of the Autism Diagnostic Observation Schedule – 2 with deaf children and young people (ADOS-2 Deaf adaptation). Validation included 122 deaf participants (aged 2–18 years), 63 with an Autism Spectrum Disorder (ASD). This was compared to a National Institute for Health and Clinical Excellence (NICE) guideline standard clinical assessment by blinded independent specialist clinicians. Results showed overall sensitivity 73% (95%CI 60%, 83%); specificity 71% (95%CI 58%, 82%), and for the more common modules 1–3 (combined as in previous studies) sensitivity 79% (95% CI 65–89%); specificity 79% (95% CI 66–89%) suggesting this instrument will be a helpful addition for use with deaf children and young people.

**Supplementary Information:**

The online version of this article contains supplementary material (10.1007/s10803-021-04931-y).

## Introduction

The median prevalence of Autism Spectrum Disorder (ASD) internationally is reported to be 0.6% (Elsabbagh et al., 2012) and approximately 1% in the UK (Baird et al., 2006). Many people with ASD require support in education and social care (Buescher et al., 2014; Knapp et al., 2009). People with an ASD are likely to have developmental differences compared to neurotypically developing people with challenges in social communication and social interaction as well as persistent repetitive patterns of behaviour, interests or activities (American Psychiatric Association (APA), 2013). They may struggle with interpretation of some social rules and social cues and how to accurately estimate the emotions or intentions of others, compared with the general population (Baron-Cohen, 2008; Hayes & Watson, 2013). They are also more likely to have unusual or intense preoccupations (Bishop et al., 2006) and to engage in repetitive actions and behaviors (Goldman et al., 2009).

The detection of ASD in deaf children as early as practicable is important to enable appropriate planning of education pathways and attuned support to parents. Many deaf children can experience a reduced exposure to language development opportunities (Hall, Levin, et al., 2017). This occurs for example when a profoundly deaf child in a hearing family has no access to either sound or signing. This can adversely impact upon executive functioning (Hall et al., 2018) and empathy skill development (Johnson et al., 2016; Peterson, O’Reilly et al., 2016a, 2016b). For example deaf children without ASD may experience delayed conversational reciprocity, delay in understanding or making accurate guesses about the feelings of others or delays in sustaining same age peer relationships. These types of problems are also seen in ASD (Bottema-Beutel et al., 2019). This overlap of developmental presentations can lead to misinterpretation in deaf children (Wright & Oakes, 2012).

Parents and carers of deaf children report having difficulty gaining an ASD assessment for their child (Young et al., 2019). Clinicians also report experiencing difficulties in the ASD assessment process with deaf children (Brenman et al., 2017) as a result of complexities in presentation of social communicative differences in deaf children that may be confused with ASD (Wright & Oakes, 2012). The reported difficulties with access to assessment (Young et al., 2019) and problems with diagnosis (Brenman et al., 2017; Roper et al., 2003) increase the risk that deaf children may not access appropriate interventions and support as early as their hearing peers. This is important since ASD research suggests early identification and intervention (e.g. appropriate educational placement) can have a positive impact on outcomes (Warren et al., 2011).

To date there are no validated diagnostic assessment instruments for ASD in deaf children (Young et al., 2019). The Diagnostic Instruments for Autism in Deaf Children Study (DIADS) set out to adapt and validate autism assessment tools for deaf children. One such instrument is the Autism Diagnostic Observation Schedule (ADOS-2), a play and interaction based assessment (Lord et al., 2012) that takes the form of a series of play and conversation-based activities between a trained assessor and the individual being assessed. The assessor seeks to provide opportunities in a standardised context for social communication, and a range of other behaviors and skills (including repetitive behaviors) to be observed and assessed. The ADOS-2 has five modules: Toddler Module, Module 1, Module 2, Module 3, and Module 4. The trained assessor selects the module that is suitable for the individual based on expressive language abilities and developmental age (Lord et al., 2012). Whilst other recent adaptions have been made to the ADOS-2 (e.g. for minimally verbal adolescents and adults) (Bal et al., 2020) this has not yet taken place for deaf individuals.

## Methods

Our study was carried out in two stages, the adaptation/modification and translation of the ADOS-2 to make it suitable for use with deaf subjects, and the initial validation study of the modified version of the ADOS-2 known as the ADOS-2 adapted for use with deaf subjects (ADOS-2 Deaf adaptation).

### Approvals

The study was carried out with the agreement of the original authors (Lord et al., 2012) and permissions from the publishers of the ADOS-2, Western Psychological Services (WPS). The Delphi Consensus Phase was approved by the sponsor on 22/05/2014 South Yorkshire. REC Reference: 15/YH/0093. We received a positive ethical opinion for the validation phase of the study from the National Research Ethics Service (NRES) Committee Yorkshire & The Humber – South Yorkshire on 17/04/2015 (reference: 15/YH/0093).

### Procedure

#### Adaptation of ADOS-2 using Delphi Consensus Method

In order to obtain the expertise of international experts in autism in deaf children we carried out a Delphi consensus process (Sharkey & Sharples, 2001). Potential participants were identified by a scoping review of the recent literature on autism in deaf children, internet searches for deaf child mental health clinical services internationally and from contacts through professional networks and international conferences, followed by snowballing techniques to identify experts (Hogan et al., 2014). Members of identified clinical teams and authors of peer-reviewed academic papers were also contacted and invited to participate in the process. Interested individuals completed a proforma and needed to have a minimum of one year’s experience of working with deaf children with ASD and to have experience of play based assessment in ASD. This ensured that they were familiar with and had used the ADOS-2 within their clinical and /or research work. These experts were mainly experienced clinicians working with deaf children with ASD, deaf clinicians and one parent. This group formed the Delphi International Expert Panel (DIEP) (see below and also Wright et al., 2020 for further details). Using their knowledge and experience, the DIEP were asked to review the content of the ADOS-2 (both activities and wording of item codings). Where these were not considered appropriate for use in deaf children, participants were asked to suggest modifications. The use of an online Delphi consensus survey platform allowed sharing of opinions and discussion about recommendations regarding which elements of the ADOS-2 required modification.

**Fig. 1:**
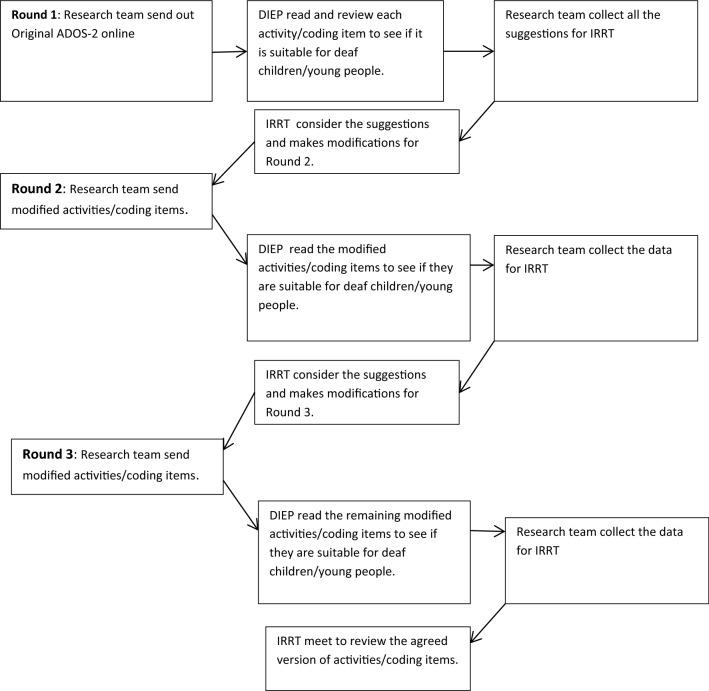
Flowchart of the Delphi consensus process with Delphi International Expert Panel (DIEP) and the Independent Research Review Team (IRRT)

The Delphi consensus was carried out over 3 iterative rounds in total (Fig. 1—Flowchart of the Delphi consensus process).

During each round the DIEP were given a series of structured questions about each ADOS-2 activity and coding item across all 5 modules, and asked to indicate whether they were suitable for use with a deaf child/young person. The options given to the DIEP are illustrated in Table 1.

**Table 1:** Delphi Consensus decision matrix for each item across all modules in the ADOS-2 assessing whether fit for purpose for use with deaf children

Item type	Options presented to Delphi participants
*ADOS-2 activity*	Yes, keep the activity the same
	Yes, although the activity needs to be modified
	No, discard the activity
*ADOS-2 Coding item*	Yes, keep the item the same
	Yes, although the item needs to be modified
	No, discard the item

If the DIEP indicated that the item needed to be modified, a text box was opened and they were asked to recommend a modification to increase the suitability of the item.

In the existing ADOS-2 assessment the trained assessor is asked to code behaviors displayed by the child/young person using specific criteria. These coding item criteria were also included in the Delphi process to ascertain whether both the codes and the guidance descriptors were appropriate for use with deaf children/young people.

Finally the DIEP were asked about the chronological benchmark criteria stipulated in the ADOS-2 coding booklet against which coding should be judged (chronological or developmental age) (see Table 2).

**Table 2:** Delphi Consensus decision matrix for coding guidance statement

Item type	Options presented to Delphi participants
Guidance statement for coding	It is appropriate to code in relation to chronological age expectations
	It is not appropriate to code in relation to chronological age expectations
	It is more appropriate to code in relation to developmental level
	It is more appropriate to code in relation to estimated expressive language skills

After each round of the Delphi-consensus the responses from the members of the DIEP were summarised and collated by the research team and presented to the Independent Research Review Team (IRRT). The IRRT included individuals with expertise in: (i) autism in deaf people and a developmental understanding of deaf children (consultant child psychiatry, consultant clinical psychology); (ii) clinical academic researchers in the field of autism; (iii), linguistics and speech and language therapy; (iv), a Deaf Clinical Consultant with lived experience of being deaf and (v) a team comprising of both Deaf and hearing researchers.

The pre-specified criteria for ‘agreement’ were those used in previous Delphi methodology consensus research (Beattie et al., 2004) advising that 80% of the DIEP needed to accept or reject the presented version of an ADOS-2 activity, the wording of coding item and the coding guidance descriptors. Where there was less than 80% agreement between DIEP members, suggested modifications were considered during IRRT meetings and a modified version of the item was then re-circulated as part of the set of structured questions in a subsequent round of the Delphi process. Fewer items were included in each round. In the third and final round, agreement of 75% or more DIEP members was required to accept the version of an activity or coding item. Previously published systematic review work has reported that 75% is the median figure used in studies of this nature, with 80% being the initial figure used by most studies (Diamond et al., 2014).

One of the pre-specified goals for the IRRT was to try to ensure that the modifications of ADOS-2 assessment activities, the wording of coding items, and the coding definitions were kept to a minimum; allowing as much conceptual integrity of the original assessment measure and coding systems to be retained as possible.

Following completion of the Delphi Consensus the modified ADOS-2 adaptation for deaf subjects with the permission of the publishers (Western Psychological Services) will be referred to as the ADOS-2 Deaf adaptation.

#### Translation of the ADOS-2 Deaf adaptation into British Sign Language

The adapted instrument was then available in written English for use with deaf people who used either spoken English or sign language as their preferred language including for example American Sign Language (ASL), British Sign Language (BSL) or Australian Sign Language (Auslan). In order to undertake the second stage of this research study – the validation of the ADOS-2 Deaf adaptation assessment procedures with deaf children and young people in the UK, the assessor scripts for each activity were translated into British Sign language (BSL). Well-established guidelines for translation between spoken and signed languages were used (Moore et al., 2013; Roberts et al., 2015). This involved strict forward translation and independent blinded back translation with reiterations until successful equivalence was achieved. To do this, four bilingual (BSL and spoken English) researchers were involved; two of whom forward translated the relevant sections from the written English ADOS-2 Deaf adaptation into BSL, and two translated the information back from BSL into written English, blind to the newly modified written English assessor scripts.

#### Validation of the ADOS-2 Deaf Adaptation

For this first validity study we used an independent blinded NICE guideline standard clinical assessment undertaken by one of 21 experienced clinicians from the UK National specialist Deaf Child and Adolescent Mental Health Service (NDCAMHS) (Wright et al., 2012), as the comparator to assess diagnostic accuracy. This clinical assessment involved taking a comprehensive history (including developmental history, family, social and medical history) from the parent and completing an ASD assessment semi-structured questionnaire using the World Health Organization (WHO) Research Diagnostic Criteria for ASD (WHO, 1993). The clinicians had the opportunity to interact and/or play with the child (usually at home but sometimes in school) to elicit possible ASD symptoms on the autism spectrum. They were also able to view screening information (both individual answers and total score) collected from the parent using a Social Communication Questionnaire (SCQ) (Chandler et al., 2007; Rutter, Bailey & Lord, 2003). Additionally, clinicians had the option to observe the child in a school setting, speak with the teacher, and give a narrative ASD questionnaire to the teacher to gather further information (Wright et al., 2016). The clinicians had access to reports from other professionals (e.g. speech and language therapy, educational psychology) made available by parents. Using this collated information, the clinicians completed a NICE guideline standard clinical assessment matrix based on the ICD 10 criteria for ASD (WHO, 1993) and gave a best estimate conclusion as to whether a diagnosis of ASD was appropriate or not. The NDCAMHS clinicians undertaking the independent NICE guideline standard assessment did not know the child or families prior to assessment and were blind to the findings of the ADOS-2 Deaf adaptation assessments and vice versa. There was no set order to the clinical assessments undertaken, which were organised according to clinician and family availability.

The ADOS-2 Deaf adaptation assessments were conducted by a group of 30 clinicians who had either received training to administer the ADOS-2 during their career with a top up training for the newly adapted ADOS-2 Deaf adaptation or they were clinicians (usually based in NDCAMHS) who had successfully completed a bespoke 5 day ADOS-2 Deaf adaptation training course. The existing ADOS-2 algorithm (Lord et al., 2012) was used with the ADOS-2 Deaf adaptation. The DIADS team provided training and supervision for the newly trained ADOS-2 Deaf adaptation assessors. Inter-rater reliability was investigated (see below). The selection of the individual ADOS-2 Deaf adaptation module was made by the trained assessors using information gained from the brief ASD parent questionnaire, discussion with the parent, information gathered from bespoke study demographic questionnaires and preliminary interaction with the child (Lord et al., 2012). Specifically close consideration was given to their preferred language and the level of expressive and receptive skills in that language in line with manual guidance (Lord et al., 2012).

### Participants

#### Inclusion Criteria

Aged between 2 and 18 years with bilateral hearing loss that was at least ‘mild’ (40 dBHL) in both ears AND they:

(i) had an existing ASD diagnosis or
(ii) had a new referral for assessment of ASD or
(iii) were not suspected to have ASD.

Deaf children and young people (and their parents/guardians) completed the assessment process using spoken English, Sign Supported English (SSE) or British Sign Language (BSL) as chosen by them.

#### Exclusion Criteria

Given high rates of co-morbidity in deaf children, subjects were not excluded on the basis of co-morbidities such as intellectual disability or other health or mental health problems.

The children were classified as having ASD or not having ASD based on a blinded NICE guideline standard clinical assessment (n = 118). In the small number of children where this was not possible (4 in total) we classified these children using either the parent report of a formal diagnosis of autism spectrum disorder from an existing professional NHS clinical assessment or if no assessment was available, by a score above the upper threshold (≥ 15) of the SCQ (a widely used screening tool for ASD with established cut-offs) (Rutter, Bailey & Lord, 2003; Chandler et al., 2007). Sensitivity analyses could therefore be conducted comparing results with the new ADOS-2 Deaf adaptation both for those who had received the NICE guideline standard clinical assessment only (n = 118) and also those of this wider *diagnostic group* (n = 122).

### Recruitment

To enable recruitment, all schools for deaf children were contacted as were all mainstream schools with specialist resource bases for deaf children and all additional educational needs schools in England. Schools were asked to circulate details of the study to the parents or guardians of potentially eligible children. Researchers were available to attend information sessions at schools where requested. The study team also contacted the 10 specialist NDCAMHS centres across England (Wright et al., 2012) asking them to circulate study information to eligible families who were currently or had previously accessed these services. Throughout the research, the study team worked closely with the NDCAMHS teams as they carried out new ASD assessments to offer interested families entry to the research alongside the assessment process. Many organisations also agreed to share study information with their members including: the National Autistic Society, National Deaf Children’s Society and the national ASD-UK and Daslne (Database of Children Autism Spectrum Disorder Living in the North East) research databases. The study was advertised on various social media platforms including Limping Chicken, an online blog aimed towards the Deaf community and various online parenting groups and platforms. Prior to taking part in the study, families were asked to complete a demographics form. One question focused on the child/young person’s preferred language/method of communication; this information was shared with assessing clinicians to ensure that they were able to identify the most suitable clinician to lead the ADOS-2 Deaf adaptation and were able to anticipate the most relevant module to use in assessment. Parents also reported clinical diagnoses (comorbidities) given to the child from NHS services.

### Analysis

Descriptive statistics are presented as mean (sd) or number (percentage). To compare ADOS-2 Deaf adaptation scores between deaf children with ASD and deaf children without ASD, a t-test was used. Diagnostic accuracy refers to the amount of agreement between the results from the diagnostic test under study (ADOS-2 Deaf adaptation) and those from a reference test (diagnostic groups—deaf with ASD and deaf without ASD) (Bossuyt, 2015). An additional sensitivity analysis was undertaken based on the blinded NICE guideline standard clinical assessment matrix only. The sensitivity and specificity were calculated based on the existing published ADOS-2 diagnostic cut-off values (Lord et al., 2012). Aspirational pre-specified criteria were—85% sensitivity and 60% specificity. We set the sensitivity high as we wanted an instrument that could correctly identify true positives and previous research on the ADOS-2 had shown sensitivity values in the range 60–95% (McCrimmon & Rostad, 2013). We pre-specified specificity in line with recently reported ADOS-2 studies (Tsheringla et al., 2014), including studies of translated versions of ADOS-2 (Medda et al., 2019).

To explore the reliability of the scoring algorithms in deaf children, Cronbach’s alpha analysis was completed to assess internal consistency. The inter-rater agreement for the ADOS-2 Deaf adaptation was assessed using Fleiss Kappa. Two experienced clinicians independently rated separate recordings of the same ADOS-2 Deaf adaptation from a subsample of recordings where parents had consented for the assessment to be videoed.

For analyses, the algorithm items and cut-off scores are those reported for the published ADOS-2 (Lord et al., 2012). A comparison score ranging from 1–10, allows amalgamation of these modules to give age adjusted scores with scores of 5–7 autism spectrum and 8–10 autism as described in the manual (Lord et al., 2012) and has been used successfully in previous publications (Shumway et al., 2012). We have combined Autism and Autism Spectrum thresholds within the ADOS-2 Deaf adaptation to define ASD.

Analysis was undertaken on STATA/SE 14.2 (Baldwin, 2019; StataCorp, 2015).

Using a standard proforma, clinicians using the ADOS-2 Deaf adaptation were asked by a research assistant to feed back to the researchers about the use of the instrument.

### Sample Size

The target sample size was 65 deaf children with ASD and 65 deaf children without ASD. This was conducted following statistical procedures described by Bland (2015) and based on estimating the difference in mean scores between deaf children with ASD and deaf children without ASD to within ± 0.34 standard deviations (95% confidence interval on each side of the estimate). For the inter-rater reliability of video recordings of the ADOS-2 Deaf adaptation, a sample size of at least 80 children was chosen to achieve 80% power to detect an intraclass correlation of 0.70, under the alternative hypothesis when the intraclass correlation under the null hypothesis is 0.50 using an F-test with a significance level of 0.05.

## Results

### Delphi Consensus Adaptation Process

Thirty five international experts (UK (44%), Australia (31%), USA (25%)) were recruited to take part in the DIEP, 16 of whom identified themselves as having expertise in the use of the ADOS-2. The majority of the DIEP were clinicians from professional background such as clinical psychology (44%) and psychiatry (19%) with others working in developmental paediatrics (12%), education (12%), speech and language therapy (6%), and CAMHS (6%). Most of the DIEP were hearing (88%) and identified spoken English as their preferred language (95%) with signed languages (Australian Sign Language (AUSLAN), American Sign Language (ASL) or BSL) making up the other five percent. Ninety four percent were female.

From the existing activities and coding items for each module in the original ADOS-2 assessment some items were considered to be fit for purpose to be used with deaf subjects and remain unchanged for all 5 modules. Fourteen activities and 51 coding items were banked without any modifications in the first round by the Delphi Consensus process. The opinions from the DIEP members were collated and presented to the IRRT where any adaptations or modifications if needed were agreed and ‘banked’ or taken into further rounds for discussion (see Table 4 for more details).

All activity/coding items were agreed within three rounds of the Delphi process with the exception of one coding item [A3 Intonation of Vocalizations or Verbalisations] in both the Toddler Module and Module 1. The IRRT and DIEP considered the wording of this item within the context of the available evidence for language development in deaf children. Following these discussions, and with agreement from the original first author, it was decided to retain the original item wording. This was because of insufficient evidence related to vocalizations in young deaf children learning language, insufficient evidence to guide clinicians to make an equivalent decision related to the development of signed language, and the original requirement to include this item within each of the module scoring algorithms. The DIEP recommended keeping the item to avoid altering the original version too greatly and to enable the researching clinicians to gather research evidence of its expression in different groups in the validation stage of the research. Table 3 outlines the Delphi agreements across rounds.

**Table 3:** ADOS-2 Deaf adaptation results illustrating the number of items agreed (banked) per module for each round

Actvities	Banked	Remaining	Total activities	Coding	Banked	Remaining	Total Codings
*Round 1*							
Toddler Module	5	10	15	Toddler Module	12	29	41
Module 1	2	8	10	Module 1	15	19	34
Module 2	3	11	14	Module 2	9	20	29
Module 3	1	13	14	Module 3	7	22	29
Module 4	3	12	15	Module 4	8	24	32
*Round 2*							
Toddler Module	15	0	15	Toddler Module	39	2	41
Module 1	10	0	10	Module 1	32	2	34
Module 2	13	1	14	Module 2	29	0	29
Module 3	6	8	14	Module 3	28	1	29
Module 4	14	1	15	Module 4	32	0	32
*Round 3*							
Toddler Module	15	0	15	Toddler Module	40	1	41
Module 1	10	0	10	Module 1	33	1	34
Module 2	14	0	14	Module 2	29	0	29
Module 3	14	0	14	Module 3	29	0	29
Module 4	15	0	15	Module 4	32	0	32

Modifications made to the ADOS-2 Deaf adaptation modules can be grouped into five categories: Structure of the instrument, Communication and language development, Cultural appropriateness, Establishing valid delivery of a test activity for deaf individuals and Knowledge/skills that clinicians need to have when assessing a deaf individual. Examples of the main changes made to activities in each category are included below.

#### Structure of the Instrument

A main recommendation in the structure for delivery of the ADOS-2 Deaf adaptation is for the deaf child/young person to be able to communicate using their preferred language during the assessment. If the child/young person preferred to use sign language, a deaf signing clinician should lead the assessment and if the child/young person preferred to communicate with spoken language, a hearing or deaf clinician (whose preferred language was spoken language) should lead the assessment. A parent/carer was present with younger children (as specified in the ADOS-2 manual for modules 1 and 2) but not present with older young people (e.g. during the administration of module 4) (Lord et al., 2012). A qualified sign language interpreter (trained in working with the ADOS-2 Deaf adaptation) was with the clinician and child/young person in all modules to make sure no communications were missed. Using this process, the clinicians are able to consider both the spoken and signed language communications, including vocalisations, gestures and prosody in both languages. This recommendation is based on the knowledge discussed in the DIEP that many children may be bilingual and may code switch (move from sign to spoken language) or code blend (use both simultaneously) (Herbert & Pires, 2017; Swanwick, 2016) and that without skilled observation this is easy to miss. The interpreter alongside the clinicians can also provide relevant information about language and communication anomalies (Ackroyd, 2018).

#### Communication and Language Development

The DIEP recommended modifications to the wording in relation to ‘Language and communication’ throughout the ADOS-2 Deaf adaptation modules in order to capture information about deaf children who may use a range of communication strategies (for example, spoken language, signed language or a mixture of signed and spoken language). The most common modification was a change to ‘words/signs’ where the original text had stated ‘words’, or ‘vocalizations/gestures’ instead of ‘vocalizations’. These changes applied to 11 activities and 65 coding items across the five modules. It was noted that features of ASD such as echolalia would occur in signs rather than words in children whose preferred language is sign language (Shield et al., 2017).

There was one coding item which the DIEP and IRRT were unable to reword to integrate appropriate parallels for signing children. For this item ‘A1a Frequency of babbling’ in the Toddler module, the overall consensus was to remove the item despite there being some emerging research on the development of a babbling equivalent in signed language (sometimes referred to as manual babbling or mabbling) (Petitto & Marentette, 1991). The judgement of the IRRT was that at the present time there was not sufficient evidence about how best to accurately code the development of babbling in a signed language. Specifically, it was considered that the difficulties distinguishing manual babbling, sign approximations, gesture and random hand and arm movements in deaf signing toddlers could lead to unreliable scoring. This item has been removed from the ADOS-2 Deaf adaptation.

During the DIEP there was considerable discussion about the use of the item that coded for use of intonation in Module 1. Whilst some DIEP members thought this coding would be a poor discriminator between ASD and those without ASD in deaf children because deaf children’s intonation is frequently reported as different from hearing children (Mora et al., 2012) emerging research under discussion on the DIEP suggested that vocal analysis and intonation could discriminate between hearing children, deaf children and children with ASD (VanDam & Yoshinaga-Itano, 2019). For this reason the DIEP and IRRT agreed to leave the original coding and noted that information gleaned from using this coding in future practice or research could yield important information about differences between groups.

#### Cultural Appropriateness

**Fig. 2:**
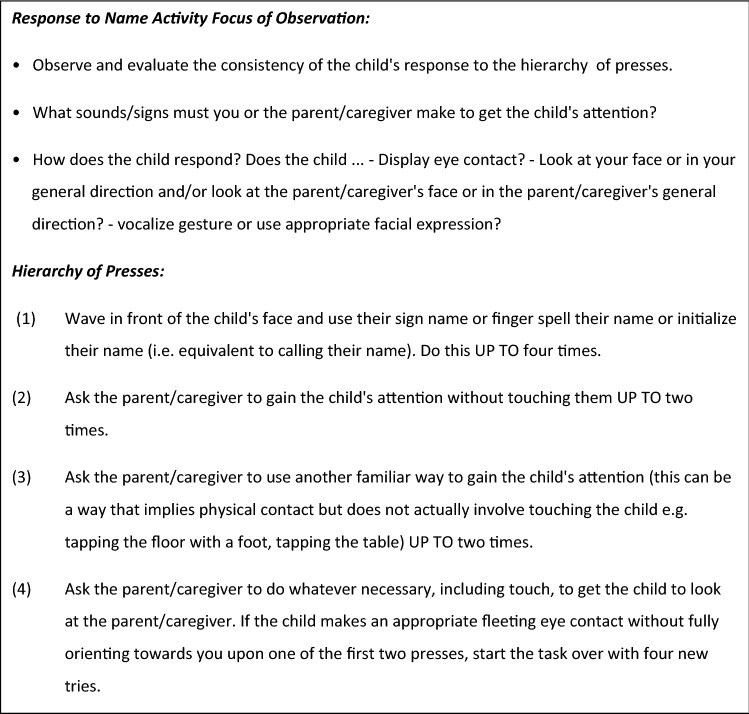
Agreed modifications to Response to Name task ADOS-2 Deaf adaptation (previously Response to Name task in ADOS-2) Toddler-module and Module 1

A cultural understanding of how for example deaf people communicate (e.g. gain each other’s attention) was considered important. Toddler Module and Module 1: the *‘Response to name’* activity was adapted after discussion of this issue. The experts considered this an inappropriate task for deaf children many of whom may be unable to hear their name called from behind (notwithstanding use of assistive hearing devices). An agreement was reached by the end of round 2 for the following modification as a task to gain the child’s attention (Fig. 2 Agreed modifications to renamed Attention task (previously *Response to Name* task)).

#### Establishing Valid Delivery of a Test Activity for Deaf Individuals

**Fig. 3:**
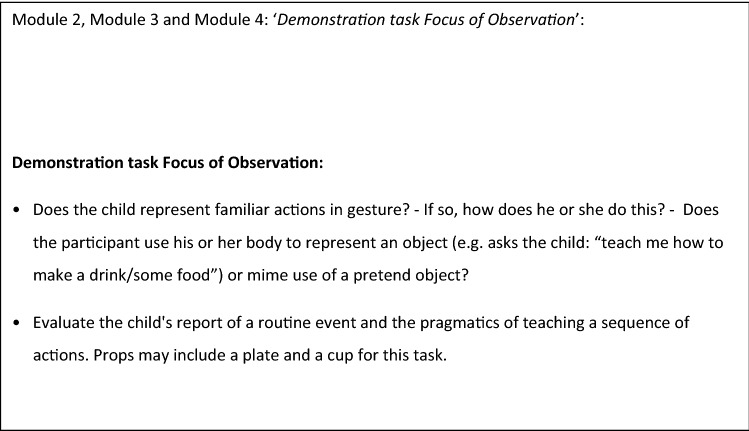
Main task modifications for Modules 2, 3 and 4—Demonstration task Focus of Observation

Additional information was incorporated in activity descriptions to ensure trained ADOS-2 Deaf adaptation assessors administered tasks using culturally appropriate ways of interacting with deaf children. Adaptations of this nature were necessary for 15 activities (with some activities appearing in more than one module). There were some activities that could not be delivered when assessing a deaf subject in the way described in the original instrument. For example the ‘*Demonstration task’* activity (Modules 2, 3 and 4) seeks to test whether a child can use gesture to communicate. In the original task the trained assessor asks the child using spoken language to demonstrate brushing teeth with the expectation that the child will attempt to use gestures to demonstrate this activity. However, in many signed languages this signed request would show the child the signs of teeth brushing which are equivalent to gestures that the test is hoping to elicit from the participant for brushing teeth. After two rounds of the Delphi process, agreement was reached with the following modifications to use a different but equivalent activity where the signed request did not show the child what to do (summarised in Fig. 3 Main task modifications for Modules 2, 3 and 4—Demonstration task).

#### Specific Clinical Knowledge/Skills

Additional information in the form of a brief addendum to the published ADOS-2 manual was suggested by the DIEP and collated and edited by the IRRT to assist ADOS-2 Deaf adaptation assessors with the administration/coding of the assessment for a deaf child/young person. These changes affect observation and coding instructions for 14 activities and 24 coding items across the 5 modules. The addendum includes information about the use of language and language approximations and highlights the importance of ensuring a language match between the lead assessor’s ability to communicate with the child (subject) in the child’s preferred language, rather than indirectly through an interpreter. This provides the child/young person the same opportunity to have direct communication with the assessor as is routinely available during assessment of hearing children. It also ensures that assessments capture cultural information that may otherwise be missed. References to this document have been included in the instructions for observations and coding within each of the modules of the ADOS-2 Deaf adaptation.

One coding item related to language abnormalities associated with autism, which appears in Modules 2, 3 and 4, has been modified to explain the features commonly present in oral children separately from the features commonly present in signing children. Details about the presentation of stereotyped/ idiosyncratic use of language in signing deaf children have been added to coding descriptions in all modules to inform clinicians about the possibilities of signing children reversing the usual direction of signs (Shield & Meier, 2012) or using space in an unusual way when signing. Similarly, modifications have been made to coding items focussed on gestures which appear in all modules (Shield et al., 2015). Information has been included to clarify that gestures are different to signs in signing children and also includes details related to the different types of gestures and when they would be expected to appear in communication with a signing child.

It was considered important for clinicians assessing deaf children to be aware of the differences between hearing and deaf children in the use of eye contact and facial expression. Modifications to coding descriptions inform clinicians that deaf children may use eye contact to engage with, or withdraw from communication and that coding should be made based on instances of flexible, appropriate use of eye contact where observed. Similarly differences in the use of facial expressions can be observed between deaf and hearing children and coding should be based on facial expressions used to communicate affective or cognitive states, not the grammatical facial expressions which are linguistic elements of signed languages.

### First Validation Study of the ADOS-2 Deaf Adaptation

Participant recruitment was completed in February 2019 and the ADOS-2 Deaf adaptation was completed with a total 122 children/young people. Blinded NICE guideline standard clinical assessment was undertaken to determine whether deaf children/young people had ASD or not (n = 118) and where this was missing for 4 children/young people using the classification described above (ASD: n = 3 scored > 15 on SCQ; without ASD n = 1). The ‘diagnostic groups’ for analysis were: 63 from the diagnostic category ‘Deaf with ASD’ and 59 from the group ‘Deaf without ASD’. Supplementary Table 1 shows the comorbidities of the two groups.

Demographic details and details of ADOS-2 Deaf adaptation module completion by diagnostic group are presented in Tables 4 and 5 respectively.

**Table 4:** Demographic characteristics

	Deaf with ASD n = 63	Deaf without ASD n = 59
*Gender*		
Male	53 (84%)	42 (71%)
Female	10 (16%)	17 (29%)
Age		
0–3	4 (6%)	9 (15%)
4–9	25 (40%)	34 (58%)
10+	34 (54%)	16 (27%)
*Ethnicity*		
White	48 (76%)	53 (90%)
Black	1 (2%)	2 (3%)
Asian	7 (11%)	2 (3%)
Mixed	6 (10%)	1(2%)
Other	1 (2%)	1 (2%)

**Table 5:** ADOS-2 Deaf adaptation module completion by group

	Deaf with ASD n = 63	Deaf without ASD n = 59	Total n = 122
Toddler module	1	1	2
Module 1	19	13	32
Module 2	6	16	22
Module 3	27	28	55
Module 4	10	1	11
Total	63	59	122

**Fig. 4:**
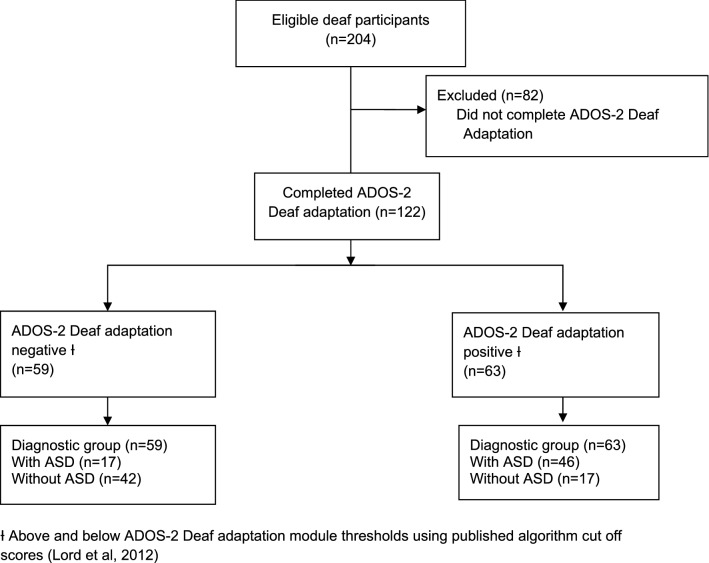
STARD Flowchart for ADOS-2 Deaf adaptation Diagnostic group. ƗAbove and below ADOS-2 Deaf adaptation module thresholds using published algorithm cut off scores (Lord et al., 2012)

The STARD flow diagram (Bossuyt, 2015) is used to show the number of participants for each diagnostic group following assessment using the modified diagnostic instrument (Fig. 4 STARD flowchart for ADOS-2 Deaf adaptation diagnostic group).

The mean scores for each module are presented in Table 6. For the combined comparison scores derived from algorithm total scores from Modules 1–3, there was a statistically significant difference between deaf with ASD and deaf without ASD (p < 0.001).

**Table 6:** Mean scores for ADOS-2 Deaf adaptation Comparison Scores *by diagnostic group

	Deaf with ASD	Deaf without ASD	Mean difference (SE), 95% CI	p value
	Mean (SD), n	Mean (SD), n		
Toddler Module: Raw total algorithm score	26, 1	2, 1	24	-
Module 1: Comparison score	7.0 (2.0), 19	1.9 (1.5), 13	5.2 (0.6), (3.8, 6.5)	< 0.001
Module 2: Comparison score	4.2 (3.5), 6	2.5 (2.0), 16	1.7 (1.2), (-0.8. 4.2)	0.179
Module 3: Comparison score	6.8 (3.0), 27	3.6 (2.5), 28	3.2 (0.7), (1.7, 4.7)	< 0.001
Combined Comparison scores (Modules 1, 2 & 3)	6.6 (2.8), 52	2.9 (2.3), 57	3.7 (0.5), (2.7, 4.6)	< 0.001
Module 4: Raw score	1.6 (2.1), 10	1.0, 1	0.6 (2.2), (-4.3, 5.5)	0.788

^*^We used published Comparison Scores (Lord et al., 2012)

There were clear group differences when using the ADOS-2 algorithm. The ADOS-2 Deaf adaptation across all modules in comparison with diagnostic group (Fig. 4) gave a sensitivity of 73% (60%, 83%) and a specificity of 71% (58%, 82%).

Supplementary Table 2 shows the sensitivity and specificity by module. When using a calibrated severity scores across modules 1–3 (Shumway et al., 2012) the combined scores (derived from ADOS-2 manual algorithm total scores) (Lord et al., 2012), compared to diagnostic group (Table 6), show a statistically significant difference between deaf with ASD and deaf without ASD (p < 0.001), and a sensitivity of 79% (95% CI 65–89%) and a specificity of 79% (95% CI 66–89%). The sensitivity analysis of the validation of the ADOS-2 Deaf adaptation against the group where group was determined by NICE Guideline standard clinical assessment alone (n = 118), gave a sensitivity of 75% (95%CI 62%, 85%) and specificity of 72% (95%CI 59%, 83%).

#### Reliability of the ADOS-2 Deaf Adaptation Scoring Algorithm

Using the raw scores from Modules 1–3, the alpha co-efficient for each module indicates high internal consistency: Module 1 (0.956), Module 2 (0.902) and Module 3 (0.887).

#### Interrater Agreement ADOS-2 Deaf Adaptation

A total of 121 assessments were filmed, of these 61 subjects did not have ASD and 60 subjects had ASD. The agreement when two clinicians coded the video blind to each other was moderate to substantial for each module on the raw ADOS-2 algorithm scores coding and using the combined comparison score (see Table 7).

**Table 7:** Inter-rater reliability for ADOS-2 Deaf adaptation

	Kappa ×	n
Module 1–3: Comparison score	0.467, p < 0.001	72
Module 1: Comparison score	0.828, p < 0.001	22
Module 2: Comparison score	0.634, p = 0.008	15
Module 3: Comparison score	0.457, p < 0.001	35

^*^The inter-rater agreement could not be assessed for the Toddler module and module 4 because of small sample sizes (n = 2 and 7 respectively)

The clinicians all of whom had experience of working within the NDCAMH service, were trained to deliver the ADOS-2 Deaf adaptation. None reported any major concerns about the administration nor the coding of this adapted measure. The only concerns raised by the trained clinicians were around the layout of the ADOS-2 Deaf adaptation module booklet with several requests to make it as similar as possible to the original ADOS-2 administration booklet and this was resolved early in the research. A final version of the ADOS-2 Deaf adaptation booklet was created and is now available with WPS publishers.

## Discussion

The Delphi consensus process achieved its aims and was considered by the IRRT to be a successful means of gathering expert opinion internationally. The DIEP remained engaged and motivated to support the development of the ADOS-2 Deaf adaptation. The administration of the new format of the assessment was also considered by clinicians using it to be a helpful tool in the assessment of deaf children.

There are some potential cost implications of the ADOS-2 Deaf adaptation in that we recommend that teams are trained, and that the clinician and a sign language interpreter are present for the interactive assessment with the clinician using the child’s preferred communication. Given the complexities of assessing deaf children it was felt that this was justifiable in that it was more likely to result in a successful assessment and an accurate diagnosis. We have no cost-effectiveness data based on follow up to substantiate this claim. Future research could examine the impact of working with qualified interpreters. It may also explore whether the use of the ADOS-2 Deaf adaptation makes assessments more readily available for deaf children, whether diagnosis appears earlier than previous findings (Roper et al., 2003) and whether outcomes for deaf children improve.

This first validation study of the ADOS-2 Deaf adaptation showed sensitivity (73%; 95%CI 60, 83%) and specificity (71%; 95%CI 58, 82%) that represent helpful results for a play and interaction based assessment. These are encouraging findings since some behaviors associated with being deaf (e.g. differences in use of facial expression and aspects of social behavior/isolation) may potentially be misinterpreted by clinicians or overlap with some of the behavioral phenotypic characteristics described in ASD. The possibility of false positives or false negatives is an important consideration when using tests of this nature and minimising misdiagnosis is particularly important in this population (Wright & Oakes, 2012) as this could lead to a range of risks or negative outcomes including inappropriate parental advice, unsuitable treatment, the child being placed in a school setting that does not meet their needs and a range of other poor outcomes.

We used the well established ICD10 criteria (WHO, 1993) for NICE guideline standard clinical assessment, widely used in Europe and beyond. This included the symptoms and behaviours within three main areas of qualitative impairment of social interaction; qualitative abnormalities in communication; and restricted, repetitive and stereotyped patterns of behaviour. These map closely to DSM5 criteria (APA, 2013) that are subsumed within two main domains of persistent problems in social communication and social interaction; alongside restricted and repetitive patterns of behaviour, interests or activities. Whilst these are operationalised slightly differently, studies using the ADOS-2 have found that DSM5 criteria may identify slightly less people as possibly having ASD than previous systems (Kent et al., 2013; Mafezsky et al., 2012; Huerta et al., 2012). Future studies could explore how different ASD criteria, including the soon to be published ICD-11, impact upon diagnosis in deaf children, but this was not the remit of the present study.

It is instructive to note that the ADOS-2 Deaf adaptation remains largely faithful to the original. Indeed we only needed to contact the original main author on two occasions for clarification or guidance.

Where modifications were made one of the main changes was in relation to the communication needs of the child in the assessment and the need to code the child by considering sign language use as an equivalent language to spoken/written languages. These modifications included careful consideration of the communication needs of the interaction itself. Whilst we have known clinicians to work directly with a sign language interpreter to engage with the child, our recommendation is for the trained assessor/clinician to be the direct participant in the interaction with the subject, meaning that for a child whose first language is sign (e.g. BSL) the ADOS-2 Deaf adaptation trained assessor needs to be fluent in that sign language. Many hearing clinicians in NDCAMHS (UK) are trained to a sign language standard (at least BSL level 3) but the DIEP recommended that BSL level 3 was not sufficient for carrying out this complex assessment with a deaf child whose first language is BSL. This is because of the large variation in language and communication use in deaf children (Swanwick, 2016) including different language abilities in both spoken and signed languages, code switching and code blending. A programme of ADOS-2 Deaf adaptation training for deaf and hearing clinicians is therefore now underway in England. The guiding principles for the delivery and conduct of the ADOS-2 Deaf adaptation state that the preferred communication needs of the deaf child/young person must be considered and that the lead clinician undertaking the assessment should have sufficient knowledge of the deaf lived experience and be able to communicate and interact directly with the child (e.g. not through a sign language interpreter).

### Strengths and Limitations

A key strength is that to date this is the largest piece of research with rigorous methodology attempting to develop and validate assessments of ASD for deaf children and young people. Researchers have previously noted the importance of bringing the deaf cultural perspective into deliberations in research (Young & Hunt, 2011). For both the DIEP and IRRT having experts with deaf lived experience enabled discussions about cultural differences between deaf and hearing experts for example in how to gain attention from each other in acceptable ways. These discussions led to specific revision recommendations.

Despite the Delphi process being online, the length of time required to review each of the ADOS-2 modules in the Delphi process was substantial. Several of the experts experienced conflicting demands on their time and mentioned struggling to find the time to complete the DIEP's successive survey questionnaire rounds. This was in part related to the constraints of the timelines due to research funding deadlines. We addressed this by giving notice well in advance of expectations to allow experts to plan their DIEP workload.

The children and young people in the study had a range of comorbidities in both those with and without ASD. This may support the likely representativeness of the sample of deaf children with a wide range of co-morbidities. Deaf children with learning disability were in both groups. Children with language delay made up a fifth of the children without ASD and nearly a third of those with ASD, which is not surprising given that language delay is common in ASD as well as being seen in deaf children. The comorbidity data needs to be treated with caution given that it is parent report. One limitation in the validity study was that it was underpowered compared to the sample size calculations. There were low numbers of participants in the toddler Module and Module 4. This is similar to reported sample sizes in other studies (Lord et al., 2012) and is likely to reflect current clinical practice in that most child ASD assessments in the UK are carried out in the age ranges that suit Module 1 – 3 (Crane et al., 2016). In this study only 13 assessments in the 0–3 years category were carried out. This relative absence of younger children brought for assessment is in part related to late presentation of deaf children as discussed earlier. Further validation research is necessary in the future to provide evidence in these developmental groups.

This first validation study has shown that the ADOS-2 Deaf adaptation provides an opportunity to observe the range of behaviours that are associated with children who have ASD in children who are deaf. These observations provide rich clinical information about behaviors such as e.g. eye contact, sharing, turn-taking and empathy skills that have occurred in a standardised context. Such observations are important for both hearing and deaf subjects and can assist discussions with parents/carers, education and care staff when considering differential diagnosis of ASD and the impact of being deaf and any other identified co-occurring physical, mental health or behavioral needs. Thus the ADOS-2 Deaf adaptation is likely to provide information that will inform both diagnostic reports and individual and family care planning.

The ADOS-2 Deaf adaptation has been culturally adapted for the assessment of deaf children so that their communication needs can be taken into account; both in the delivery and the scoring of the assessment. In this first validation study of the modified ADOS-2 Deaf adaptation we have shown that we were able to deliver the assessment in the child’s preferred language- i.e. in spoken language (in this case spoken English) for deaf children using spoken language; or in a signed language (in this case British Sign Language—BSL) with a trained assessor who communicates in sign language for deaf children using a signed language. The presence of an interpreter (or a bilingual professional) sitting with the clinician is a departure from the traditional ADOS-2 manual. We believe that this adds considerably to the quality of information gathered although this would need to be researched separately. We found no evidence of disruption to interactions as many deaf children are used to having interpreters in their daily school environment and the interpreter did not enter the interaction, although we did not specifically research this. Validation results presented here are from this delivery context. Further investigation will be necessary to replicate these findings in the assessment of deaf subjects using other spoken and signed languages.

In a separate qualitative study conducted by independent researchers who undertook qualitative interviews with a subsample of parents, they reported that parents highly valued this approach to diagnostic assessment and felt that their children appeared relaxed and seemed to enjoy the assessment (Young et al., 2019). It is hoped that this modified version of this internationally recognised standardised play and activity based assessment if shown to be reliable and valid in different languages, will provide relevant structure to the direct observation aspect of the assessment process for deaf children.

We believe that the sensitivity and specificity results are good enough to recommend the ADOS-2 Deaf adaptation for use of Modules 1 – 3, with deaf children, with this study being carried out in the UK. No adaptation to the existing algorithm has been suggested although independent future replication would be helpful. Indeed we believe that using the same algorithm is advantageous by not creating unnecessary differences from the original ADOS-2. We plan to provide further bespoke training to the ten NHS England funded child mental health centres for deaf children (NDCAMHS) so that assessors can carry out the ADOS-2 Deaf adaptation as part of the multidisciplinary ASD diagnostic assessments to a high degree of reliability, and in the child’s preferred language.

We encourage and support research to test this newly modified and validated assessment instrument in other countries. There is also a need for additional studies to investigate the psychometric properties of the ADOS-2 Deaf adaptation in samples of younger children under the age of 30 months and older adolescents and adult samples appropriate for the toddler module and module 4 respectively.

## Supplementary Information

Below is the link to the electronic supplementary material.

Electronic supplementary material 1 (DOCX 26 kb)

Electronic supplementary material 2 (DOCX 16 kb)
