# Supplementary material for: Adapting and validating the Autism Diagnostic Observation Schedule Version 2 for use with deaf children and young people
Source: J Autism Dev Disord. 2021 Mar 24;52(2):553–68. doi: 10.1007/s10803-021-04931-y (PMC8813800; doi:10.1007/s10803-021-04931-y)
Supplement: Supplementary file 1 — Electronic supplementary material 1 (DOCX 26 kb) [file 10803_2021_4931_MOESM1_ESM.docx]

**Supplementary Table 1 – Co-morbidities of participants in ADOS-2 Deaf adaptation validation study**

| **Clinical diagnoses*** | **Deaf Children**  **with ASD (n=63)**  **n (%)** | **Deaf Children without ASD (n=59)**  **n (%)** |
| --- | --- | --- |
| **Genetic** |  |  |
|  |  |  |
| Alport syndrome | 1 (2) | 1 (2) |
| Branchio-oto-renal syndrome | 0 (0) | 2 (3) |
| Chromosome 1q21.1 duplication syndrome | 1 (2) | 0 (0) |
| Chromosome 15 deletion | 0 (0) | 1 (2) |
| Connexin 26 | 3 (5) | 4 (7) |
| Mondini dysplasia | 1 (2) | 0 (0) |
| Pendred syndrome | 2 (3) | 2 (3) |
| X linked Stapes Gusher Syndrome | 0 (0) | 1 (2) |
| Waardenburg | 2 (3) | 4 (7) |
| XXY (Klinefelter syndrome) | 1 (2) | 0 (0) |
|  |  |  |
| **Developmental** |  |  |
| Dyspraxia/motor co-ordination disorder | 5 (8) | 4 (7) |
| Learning Disability | 14 (23) | 7 (12) |
| Language Delay | 18 (30) | 13 (22) |
| Socio-emotional developmental delay | 0 (0)^+^ | 2 (3) |
| Von Hippel-Landau disease | 1 (2) | 0 (0) |
|  |  |  |
| **Neurological/physical** |  |  |
| Asthma | 6 (10) | 2 (3) |
| Aural atresia | 1 (2) | 0 (0) |
| Disorder of Vestibular Function | 0 (0) | 2 (3) |
| Cerebral palsy | 0 (0) | 2 (3) |
| Past Cytomegalovirus infection | 4 (7) | 1 (2) |
| Epilepsy | 1 (2) | 3 (5) |
| Hypermobility | 1 (2) | 0 (0) |
| Microcephaly | 0 (0) | 2 (3) |
| Multiple physical problems | 5 (8) | 1 (2) |
| Sensory processing disorder | 6 (10) | 4 (7) |
| Visual problems (more than acuity) | 1 (2) | 2 (3) |
|  |  |  |
| **Mental health** |  |  |
| Attention Deficit Hyperactivity Disorder | 5 (8) | 4 (7) |
| Conduct/Behaviour | 1 (2) | 3 (5) |
| Emotional (serious anxiety disorders) | 6 (10) | 6 (10) |

*Parent reported clinical diagnoses

+all parents of children with ASD described socio-emotional developmental delay but this was not given as a specific diagnosis as in the deaf children without ASD.
