# Supplementary material for: Adapting and validating the Autism Diagnostic Observation Schedule Version 2 for use with deaf children and young people
Source: J Autism Dev Disord. 2021 Mar 24;52(2):553–68. doi: 10.1007/s10803-021-04931-y (PMC8813800; doi:10.1007/s10803-021-04931-y)
Supplement: Supplementary file 2 — Electronic supplementary material 2 (DOCX 16 kb) [file 10803_2021_4931_MOESM2_ESM.docx]

**Supplementary Table 2: Validation of ADOS- 2 Deaf Adaptation by diagnostic group**

|  | Area  Under the Curve (AUC)  (95% CI) | N  (Deaf with ASD /Deaf without ASD) | Cut-offƗ | Sensitivity | Specificity |
| --- | --- | --- | --- | --- | --- |
| Toddler Module: Raw score  All younger/older with few words  Older with some words |  | 2  1/0  0/1 | 10  8 | 100%  100% | 100%  100% |
| Module 1: Comparison score | 0.970  (0.921, 1.000) | 32  (19/13) | 4 | 89%  (67%, 99%) | 92%  (64%, 100%) |
| Module 2: Comparison score | 0.589  (0.266, 0.911) | 22  (6/16) | 4 | 50%  (12%, 88%) | 81%  (54%, 96%) |
| Module 3: Comparison score | 0.779  (0.652, 0.906) | 55  (27/28) | 5 | 78%  (58%, 91%) | 71%  (51%, 87%) |
| Combined comparison scores (Module 1, 2 and 3) | 0.822  (0.740, 0.905) | 109  (52/57) | 5 | 79%  (65%, 89%) | 79%  (66%, 89%) |
| Module 4: Raw score |  | 11  (1/10) | Algorithm | 100% | 100% |
|  |  |  |  |  |  |

Ɨ cut-offs are those for the original published ADOS- 2 (Lord et al 2012)
